# Supplementary material for: Time-varying stimuli that prolong IKK activation promote nuclear remodeling and mechanistic switching of NF-κB dynamics
Source: Nat Commun. 2025 Aug 8;16:7329. doi: 10.1038/s41467-025-62837-0 (PMC12334592; doi:10.1038/s41467-025-62837-0)
Supplement: Supplementary file 1 — Supplementary Information [file 41467_2025_62837_MOESM1_ESM.pdf]

Supplementary Information

Time-varying stimuli that prolong IKK activation promote nuclear remodeling and mechanistic switching of NF-κB dynamics

Authors

Steven W. Smeal[1], Chaitanya S. Mokashi[1,2], A. Hyun Kim[1], P. Murdo Chiknas[1], Robin E. C. Lee[1,3,4,†]

Affiliations

[1]Department of Computational and Systems Biology, School of Medicine, University of Pittsburgh, Pittsburgh, PA 15213, USA
[2]current address Altos Labs, Redwood City, CA, 94065, USA
[3]Center for Systems Immunology, School of Medicine, University of Pittsburgh, Pittsburgh, PA 15213, USA
[4]Department of Physics and Astronomy, University of Pittsburgh, Pittsburgh, PA 15260, USA
[†]Corresponding Author: robinlee@pitt.edu

Supplementary figures

Supplementary Figure 1: Dynamic stimulation device and cell responses to a single pulse

A. Schematic of the microfluidic chip as designed in the Autodesk Fusion 360 software (v2.0.21487) highlighting the dimensions of the chip for the X, Y, and Z axis, units in mm. **B.** Image of the microfluidic chip with PDMS attached to a glass cover slide, coin for scale. Green food coloring was imaged to highlight the inlets, chamber, and outlets for image purposes only. Scale bar is 20mm. **C.** Detailed pulse profiles generated by the microfluidic system for the 1x6-minute and 4x1.5-minute conditions. Alexa Fluor 647–conjugated BSA was included in the media and imaged every 5 seconds to capture high-resolution pulse dynamics. **D.** Bar plot illustrates the consistency of the area under the curve (AUC) for each pulse trajectory shown in panel C. **E.** Nuclear RelA export scores (see methods) for experimental trajectories of varying single pulse duration. A Student's two-sided t-test comparison revealed statistically significant differences ($p < 0.01$) for all groups, except between the 2- and 6-minute pulse durations, where no significant difference was observed. Single cell n values are: 0.5 min = 18; 2 min = 20; 6 min = 28; 15 min = 22; 30 min = 21. Source data are provided as a Source Data file.

Figure S1

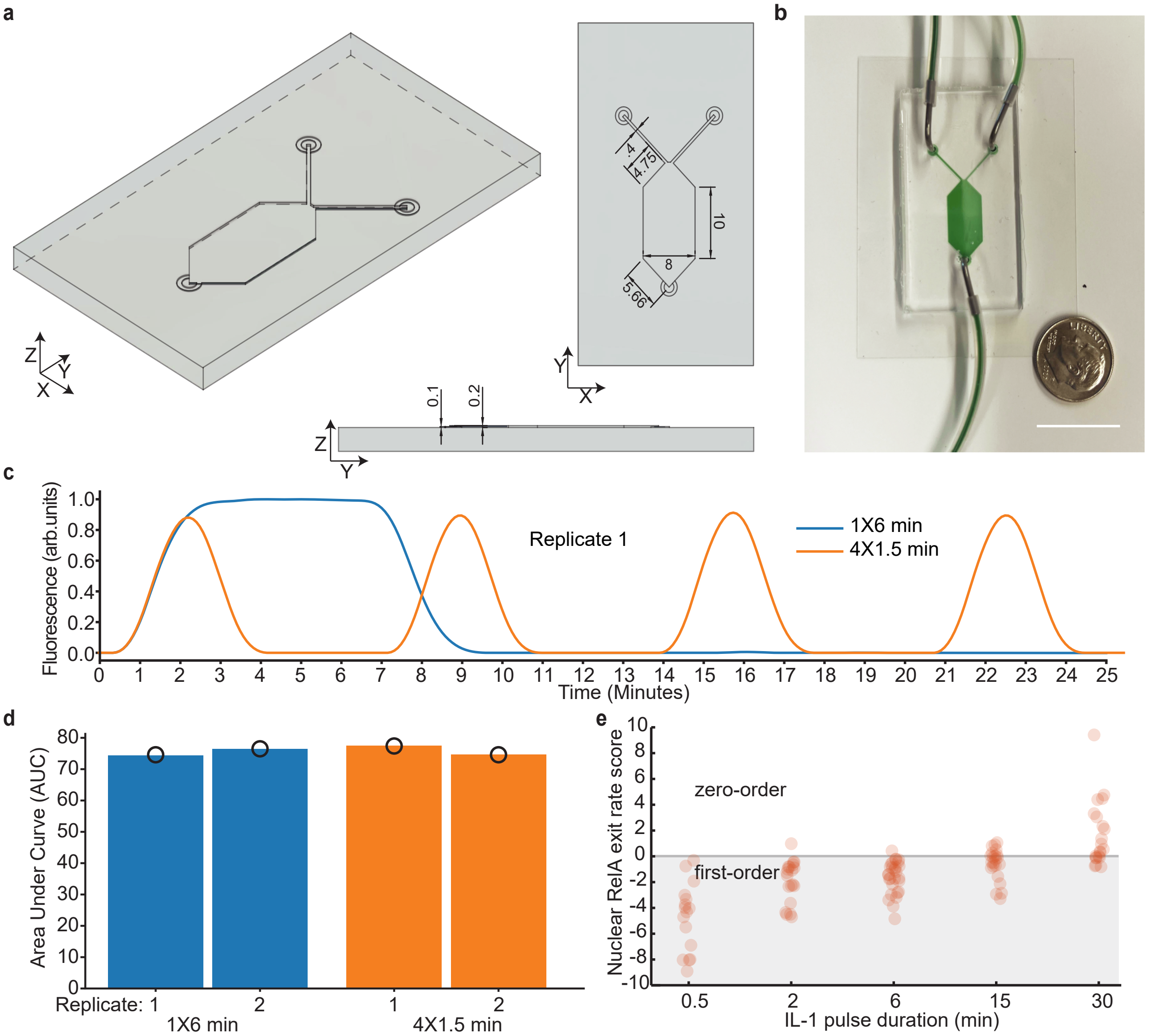

a
b
c
d
e
Z
Y
X
4
4.75
10
8
5.66
0.1
0.2
Fluorescence (arb.units)
1.0
0.8
0.6
0.4
0.2
0.0
0 1 2 3 4 5 6 7 8 9 10 11 12 13 14 15 16 17 18 19 20 21 22 23 24 25
Time (Minutes)
Replicate 1
1X6 min
4X1.5 min
Area Under Curve (AUC)
80
70
60
50
40
30
20
10
0
Replicate: 1
2
1
2
1X6 min
4X1.5 min
Nuclear RelA exit rate score
10
8
6
4
2
0
-2
-4
-6
-8
-10
zero-order
first-order
0.5
2
6
15
30
IL-1 pulse duration (min)

Supplementary Figure 2: NF-kB responses to a dose-conserving cytokine pulse train are enhanced for two and three pulse stimuli.

A. Boxplots of single-cell experimental time courses showing AUCs of NEMO spots (left) and Nuclear RelA (right). A 5-minute gap time between three 2-minute pulses of IL-1 at 10 ng/mL causes a significant enhancement of nuclear RelA. Increasing the gap time beyond 5-minutes has diminishing returns. Single cell n values are: 0 min = 28; 5 min = 23; 10 min = 16; 15 min = 16. Exact p-values as indicated for student's two-sided t-test. **B.** Boxplots of single-cell experimental time courses as in panel A for two 3-minute pulses. Increasing the gap duration up to 15 minutes enhances the AUC of nuclear RelA. Boxplots show the median value and interquartile ranges. Single cell n values are: 0 min = 28; 5 min = 19; 10 min = 12; 15 min = 21; 20 min = 17. Exact p-values of two-sided student's t-test for pairwise comparisons are: 0 min vs. 10 min (NEMO) = 0.01; 0 min vs. 5 min (RelA) = 10^{-4}; 0 min vs 10 min (RelA) = 10^{-3}; 0 min vs. 15 min (RelA) = 10^{-8}. Source data are provided as a Source Data file.

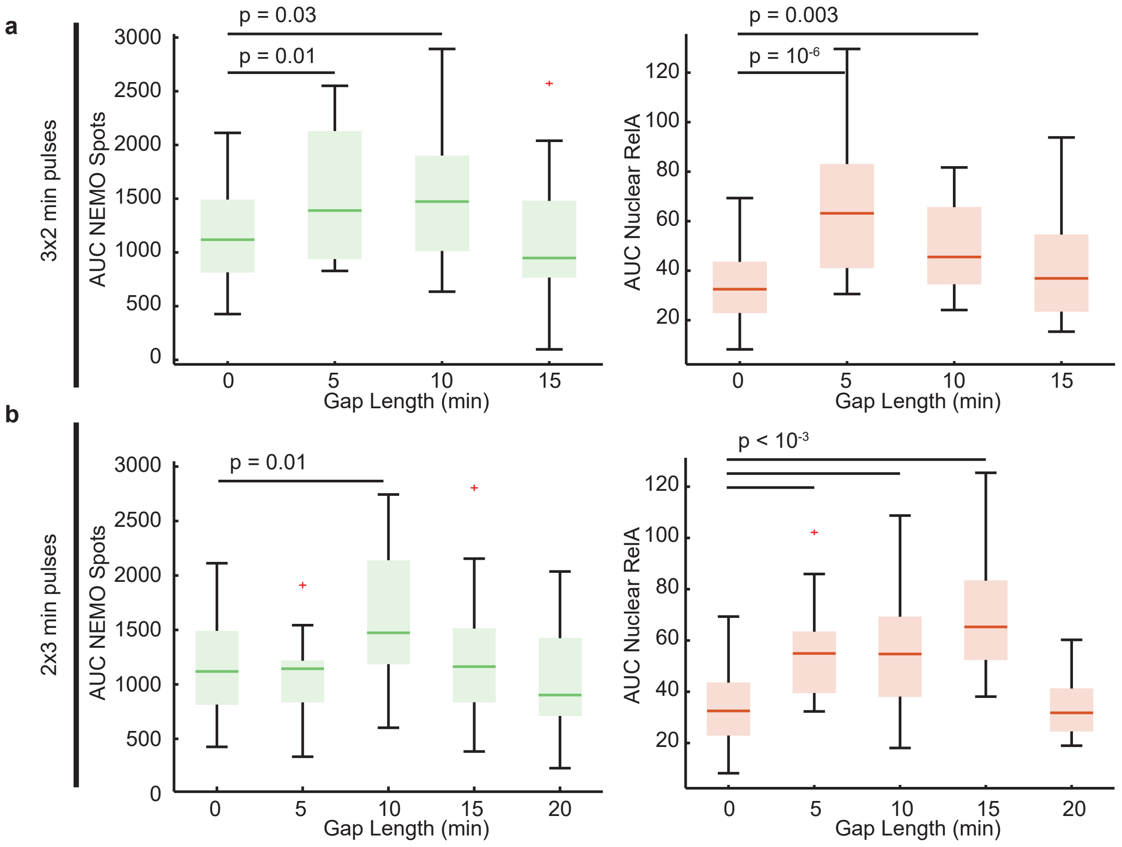
a
3x2 min pulses
AUC NEMO Spots
p = 0.03
p = 0.01
Gap Length (min)
AUC Nuclear RelA
p = 0.003
p = 10-6
b
2x3 min pulses
p = 0.01
p < 10-3

Supplementary Figure 3: KYM-1 cells exposed to a pulse train of TNF enhances the NF-kB response

A. Time lapse images of KYM-1 stably expressing mVen-RelA fusion exposed to TNF at 1 ng/mL as a single 6-minute pulse (top) or two 3-minute pulses with a 9-minute gap (bottom). **B.** Single cell trajectories of the intensity of nuclear RelA (fold change) for KYM-1 cells exposed to a continuous (left), single 6-minute (middle), and two 3-minute (right) pulses of TNF at 1 ng/mL. Orange bold lines represent mean and shaded region represents +/- 1 standard deviation of single cell trajectories. Single cell n values are: Continuous = 25; 1x6 = 29; 2x3 = 19. **C.** Box plots of the AUC of nuclear RelA (fold change) for varying the gap length of two 3-minute pulses (left) and two 2-minute pulses. Single cell n values for 1 ng/mL TNF stimulation are: Continuous = 25; 1x6 = 29; 2x3 gap 9 = 19; 2x3 gap 12= 24; 2x3 gap 15 = 20; 2x3 gap 18 = 28. Single cell n values for 0.5 ng/mL TNF stimulation are: Continuous = 27; 1x4 = 38; 2x2 gap 8 = 53; 2x2 gap 13 = 33; 2x2 gap 18 = 19; 2x2 gap 23 = 48. Exact p-values for two-sided student's t-test for pairwise comparisons of 1 ng/mL TNF are: Continuous vs. 1x6 = 10^{-8}; 1x6 vs. 2x3 gap 9 = 0.001; 1x6 vs. 2x3 gap 12 = 0.03. Exact p-values for two-sided student's t-test for pairwise comparisons of 0.5 ng/mL TNF are: Continuous vs. 1x4 = 10^{-9}; 1x4 vs. 2x2 gap 8 = 10^{-9}; 1x4 vs. 2x2 gap 13 = 0.003. Boxplots show the median value and interquartile ranges. Source data are provided as a Source Data file.

Figure S3

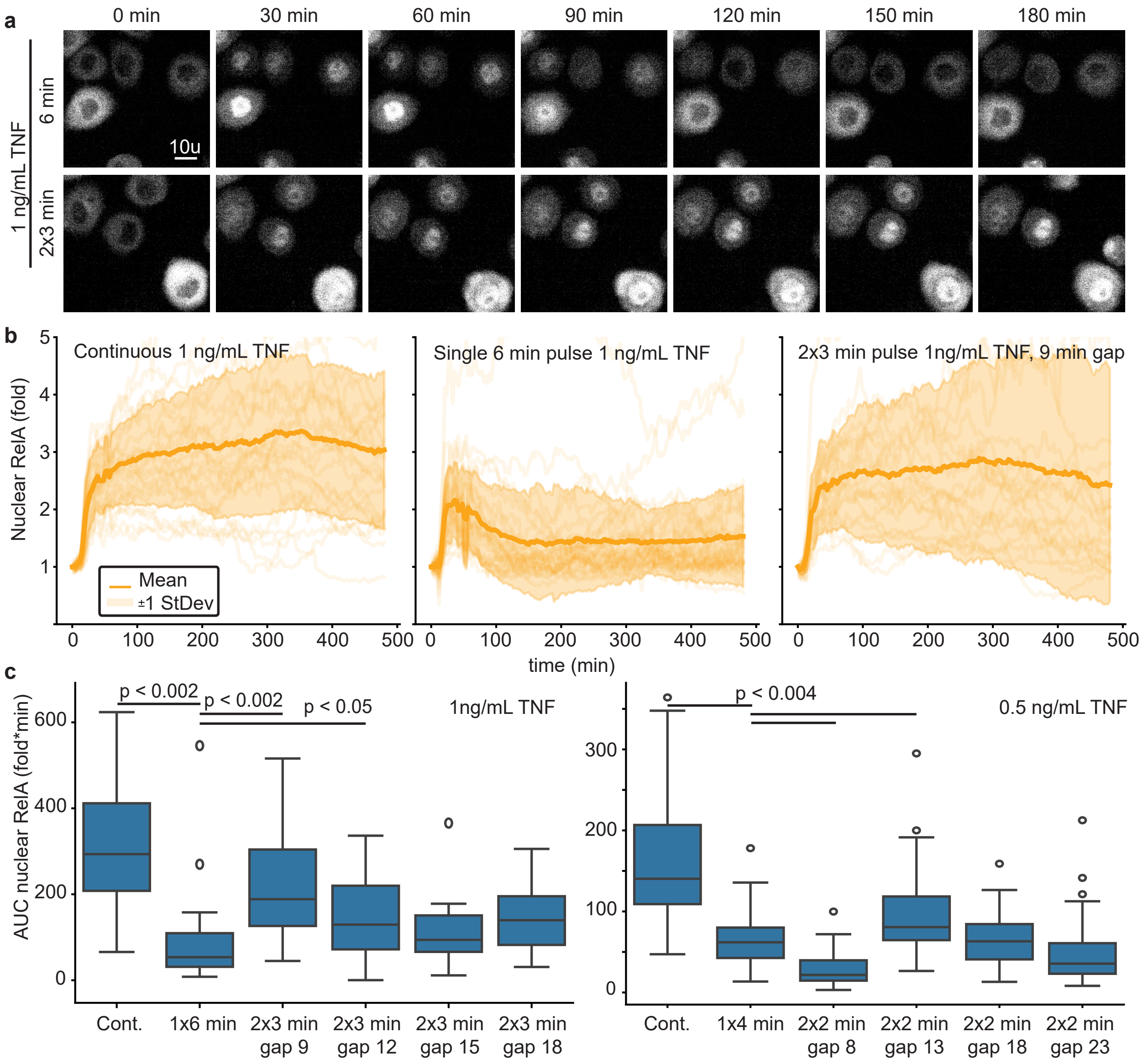

a
0 min
30 min
60 min
90 min
120 min
150 min
180 min
1 ng/mL TNF
6 min
2x3 min
10u
b
Continuous 1 ng/mL TNF
Single 6 min pulse 1 ng/mL TNF
2x3 min pulse 1ng/mL TNF, 9 min gap
Nuclear RelA (fold)
Mean
±1 StDev
time (min)
c
AUC nuclear RelA (fold*min)
p < 0.002
p < 0.002
p < 0.05
1ng/mL TNF
Cont.
1x6 min
2x3 min gap 9
2x3 min gap 12
2x3 min gap 15
2x3 min gap 18
p < 0.004
0.5 ng/mL TNF
Cont.
1x4 min
2x2 min gap 8
2x2 min gap 13
2x2 min gap 18
2x2 min gap 23

Supplementary Figure 4: Dose-conserving pulse trains switch the export mechanism of nuclear RelA to a pseudo-zero-order process.

A. Box plot of the maximum nuclear RelA (fold change) of single cell trajectories given 10 ng/ml IL-1 for single and multiple short pulses. Although significant for 2 of the 5 conditions, the trend of increased nuclear RelA is only very subtle and does not explain the enhanced AUC in response to a cytokine pulse train. Student's two-sided t-test p-values for pairwise comparisons are: 1/6 vs. 3/6 = 0.002; 1/6 vs. 4/6 = 0.004. **B.** Example trajectories highlighting the exit rate score, used to distinguish first-order and zero-order export kinetics. Experimental data are representative single cell trajectories (solid red line) and dashed lines represent models of best fit for zero and first order decay. Positive scores represent zero-order export kinetics, and negative scores represent first-order kinetics. See methods for details. **C.** Jitter plot of exit rate scores for individual cell trajectories of the single- and multi-pulse IL-1 stimulation patterns (top). Pairwise comparisons indicate p-value for student's t-test (bottom). **D.** Nuclear RelA (fold change) single cell trajectories for the 1X6 (N=28) minute and 4X1.5 (N=27) minute pulse trajectories where bold line represents the population average. **E.** Nuelar RelA exit rate scores for single cell trajectories in panel D. Source data are provided as a Source Data file.

Figure S4

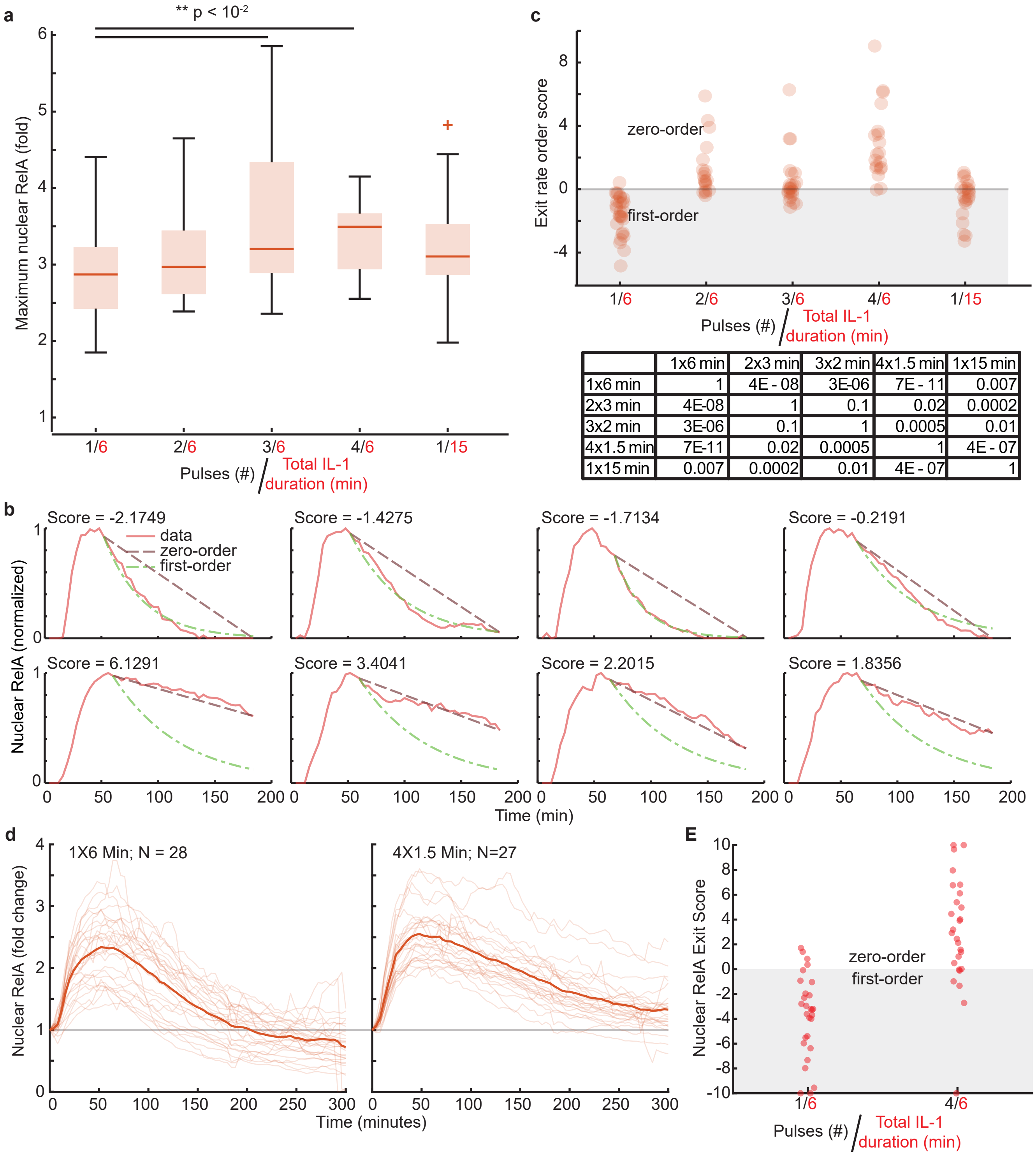

	1x6 min	2x3 min	3x2 min	4x1.5 min	1x15 min
1x6 min	1	4E - 08	3E-06	7E - 11	0.007
2x3 min	4E-08	1	0.1	0.02	0.0002
3x2 min	3E-06	0.1	1	0.0005	0.01
4x1.5 min	7E-11	0.02	0.0005	1	4E - 07
1x15 min	0.007	0.0002	0.01	4E - 07	1

Supplementary Figure 5: Original D2FC and optimized parameterizations do not recapitulate the emergent property.

A and B. Box plots comparing the D2FC simulated results of the AUC nuclear RelA of the single (A) and multi-pulse (B) stimulation pattern. D2FC model is using original parameter set and D2FC optimized is the overall best set of parameters found from the particle swarm optimization. Student's two-sided t-test p-values for pairwise comparisons are: 0 D2FC Opt. vs. Exp. = 0.001; 2 D2FC Opt. vs. Exp. = 0.02; 6 D2FC Opt. vs. Exp. = 10^{-4}; 15 D2FC Opt. vs. Exp. = 0.02; 2/6 D2FC vs. Exp. = 10^{-6}; 3/6 D2FC vs. Exp. = 10^{-4}; 4/6 D2FC vs. Exp. = 10^{-6}; 2/6 D2FC Opt. vs. Exp. = 0.01; 4/6 D2FC Opt. vs. Exp. = 0.004. The D2FC recapitulates responses to a single pulse, but not to pulse trains. Remarkably, the optimized D2FC fails to recapitulate most conditions. The relative failure of the optimized D2FC is because PSO using the emergent property (fits to averages from four selected conditions: control, 1x0.5-min, 4x1.5-min, 1x30-min) as an objective function effectively distributed the error across conditions, resulting in a model that marginally improves responses to a pulse train at the expense of even poorer fits to other conditions. **C.** Results of the D2FC, D2FC optimized, and $D2FC^2$ comparing simulated results (solid lines) with the averages of single-cell trajectories (circles) in each of three conditions. Simulation results for the D2FC optimized, and $D2FC^2$ are for lowest-error parameterizations following particle swarm optimization. **D-G** Bar graphs showing the fraction of single cell trajectories estimated express zero order exit rate dynamics for the experimental data, and model simulation of the naïve D2FC, Optimized D2FC, and $D2FC^2$. Source data are provided as a Source Data file.

Figure S5

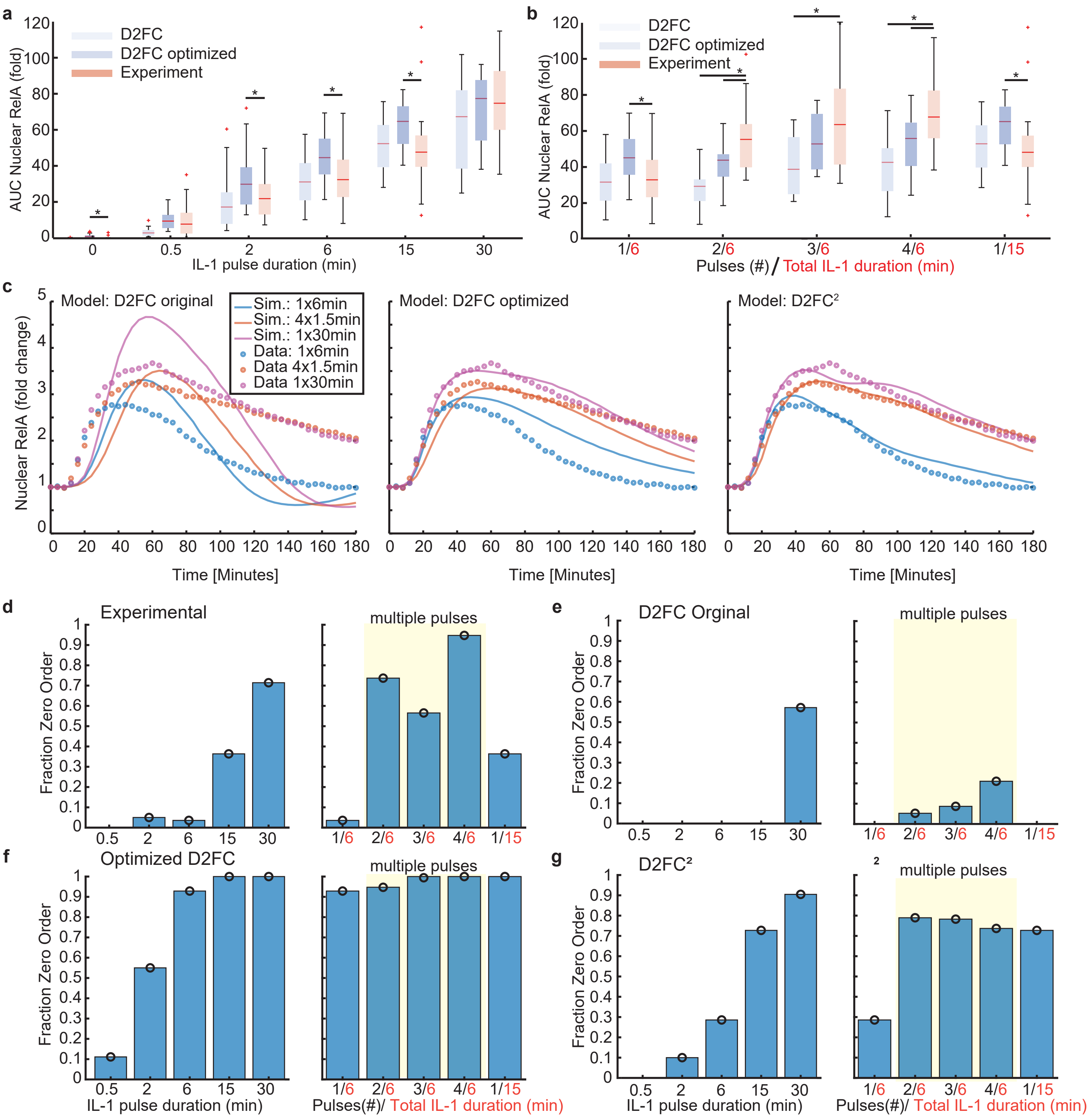
a
AUC Nuclear RelA (fold)
D2FC
D2FC optimized
Experiment
IL-1 pulse duration (min)
b
Pulses (#) / Total IL-1 duration (min)
c
Model: D2FC original
Model: D2FC optimized
Model: D2FC²
Sim.: 1x6min
Sim.: 4x1.5min
Sim.: 1x30min
Data: 1x6min
Data 4x1.5min
Data 1x30min
Nuclear RelA (fold change)
Time [Minutes]
d
Experimental
multiple pulses
Fraction Zero Order
e
D2FC Orginal
f
Optimized D2FC
g
D2FC²
IL-1 pulse duration (min)
Pulses(#)/ Total IL-1 duration (min)

Supplementary Figure 6: Post-hoc analysis of particle swarm optimization results for the D2FC^2.

A. Box plots of the parameters identified in 500 iterations of the D2FC^2 optimization using particle swarm. The y-axis is normalized relative to the parameter limits to account for differences in parameter orders. Importantly, for most parameters, there were solutions following particle swarm optimization that used the full range of values from the prior distribution suggesting that the parameter space has been effectively swarmed. **B.** Heat map of parameters for the top 10 models for the D2FC^2 normalized to the explored parameter space. Colorbar represents the normalized parameter value relative to the prior distribution (Supplementary Table 4). For most parameters there is variance between the models indicating that comparable results can be achieved via distinct parameter combinations. Source data are provided as a Source Data file.

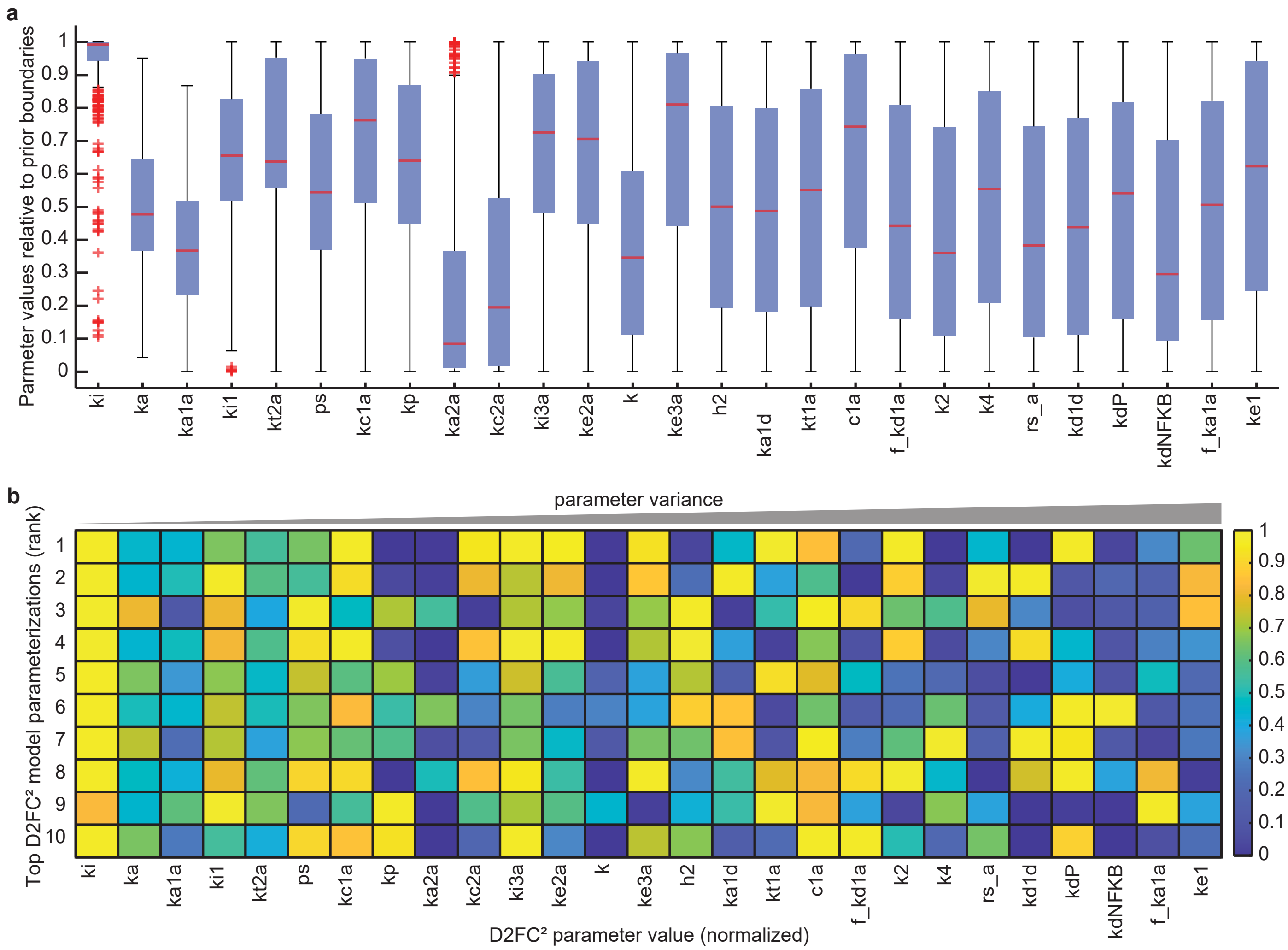
a
Parameter values relative to prior boundaries
1
0.9
0.8
0.7
0.6
0.5
0.4
0.3
0.2
0.1
0
ki
ka
ka1a
ki1
kt2a
ps
kc1a
kp
ka2a
kc2a
ki3a
ke2a
k
ke3a
h2
ka1d
kt1a
c1a
f_kd1a
k2
k4
rs_a
kd1d
kdP
kdNFKB
f_ka1a
ke1
b
parameter variance
Top D2FC² model parameterizations (rank)
1
2
3
4
5
6
7
8
9
10
D2FC² parameter value (normalized)
1
0.9
0.8
0.7
0.6
0.5
0.4
0.3
0.2
0.1
0

Supplementary Figure 7: Models testing alternative mechanisms with additional feedback inhibitors do not recapitulate the emergent property

A. Schematic of the different IkB feedback mediators tested to fit the observed emergent property. **B.** Boxplot of the error to the emergent property score of the top 50 parameterization based off the error to the training, validation, and emergent property score. IkBβ and IkBε models were run on average 367 times through the particle swarm optimization algorithm. **C.** Results of PSO from top 50 models for each of the different model architectures tested. Each model parameterization is evaluated based on the sum of squared error (SSE) to the 4 training conditions (x-axis), to the SSE of averages from the 5 validation conditions not used for PSO (y-axis), and emergent property error score (colorbar). D2FC optimized models were limited to the quadrant highlighted in pink. Source data are provided as a Source Data file.

Figure S7

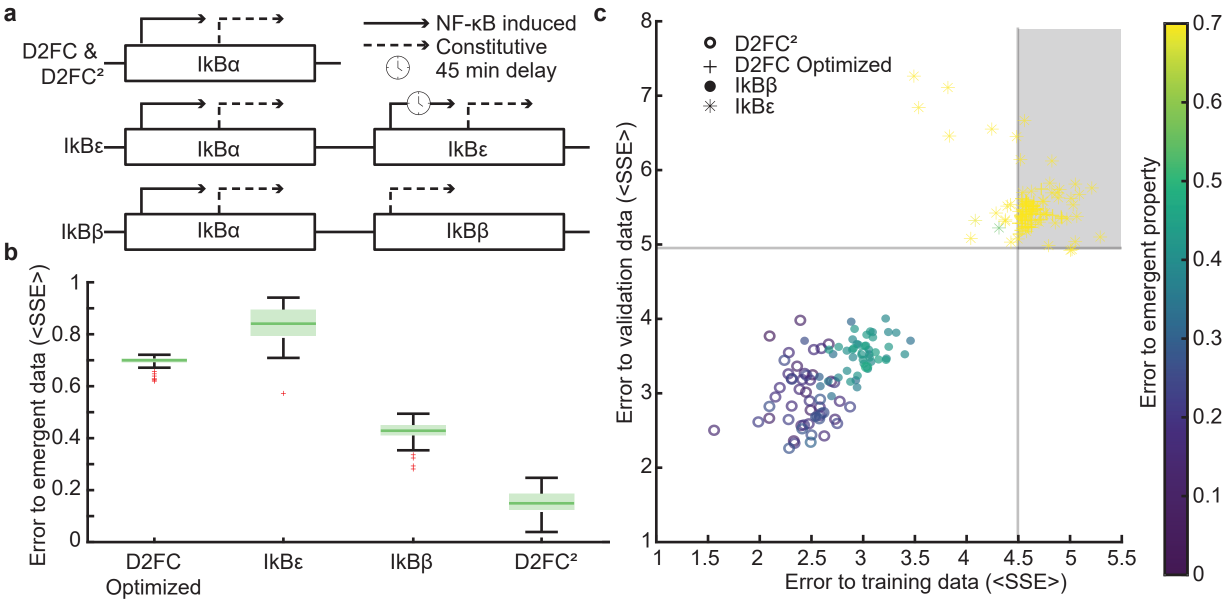

a
D2FC &
D2FC²
IkBα
NF-κB induced
Constitutive
45 min delay
IkBε
IkBα
IkBε
IkBβ
IkBα
IkBβ
b
Error to emergent data (<SSE>)
D2FC
Optimized
IkBε
IkBβ
D2FC²
c
D2FC²
D2FC Optimized
IkBβ
IkBε
Error to validation data (<SSE>)
Error to training data (<SSE>)
Error to emergent property

Supplementary Figure 8: Quality of fit to single cell trajectories for the top D2FC^2 model parameterizations.

A. Elbow plot showing the fraction of non-acceptable fits as a function of the threshold for defining an acceptable single-cell trajectory. The elbow point, mathematically identified as the point furthest from a linear line connecting the first and last values (see Methods), was used to set a threshold between high- and poor-quality fits. A strict threshold of half the elbow point was applied to identify single cell simulations fits that are considered excellent.

B. Proportion of single-cell predictions classified as excellent, high, or low quality compared to experimental data across different dosing schemes for the best-performing model. Source data are provided as a Source Data file.

Figure S8

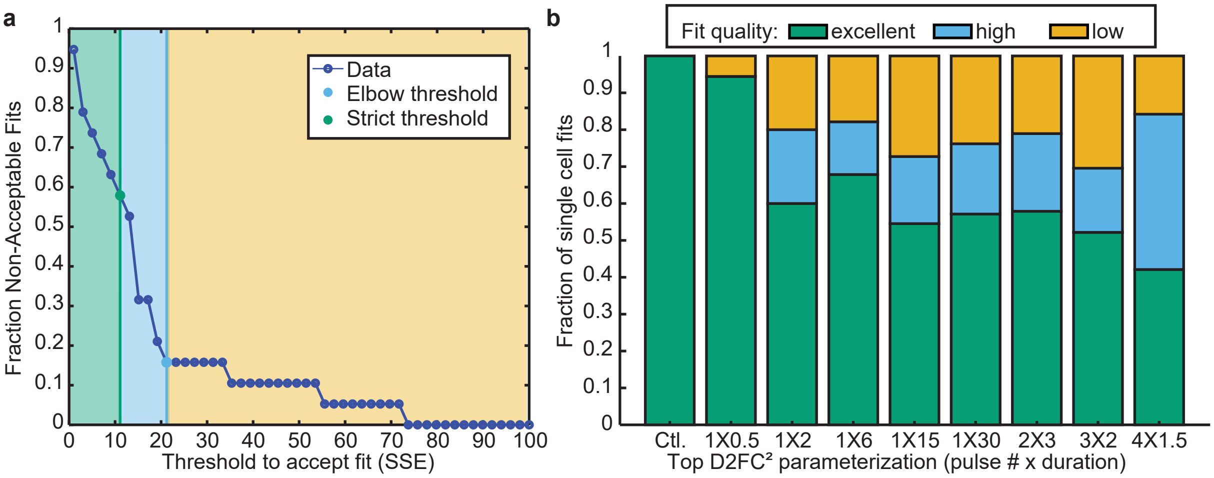

a
Fraction Non-Acceptable Fits
Threshold to accept fit (SSE)
Data
Elbow threshold
Strict threshold
b
Fit quality:
excellent
high
low
Fraction of single cell fits
Ctl.
1X0.5
1X2
1X6
1X15
1X30
2X3
3X2
4X1.5
Top D2FC² parameterization (pulse # x duration)

Supplementary Figure 9: Additional support for the FRAP and H3K4me3 analysis

A. Average recovery curves of the total immobilized where the shaded region represents the +/- 1 standard error of the mean of individual FRAP recovery curves. **B.** Boxplot summarizing replicate data H3K4me3 immunofluorescence intensity across conditions. Single cell n values are: Control = 168; 1x6 60 min = 141; 4x1.5 60 min = 182; 1x6 180 min = 143; 4x1.5 180 min = 143; 4x1.5 OICR = 176. Student's two-sided t-test p-values for pairwise comparisons are: 1x6 vs 4x1.5 60 min = 10^{-9}; 1x6 vs. 4x1.5 180 min = 10^{-4}; 1x6 vs. 4x1.5 OICR = 10^{-40}; 4x1.5 vs 4x1.5 OICR 180 min = 10^{-59}. **C.** Boxplot summarizing changes in the total immobile fraction following 4X1.5 min IL-1 with and without MLL1 inhibitor OICR-9429 do not show significant differences. Source data are provided as a Source Data file.

Figure S9

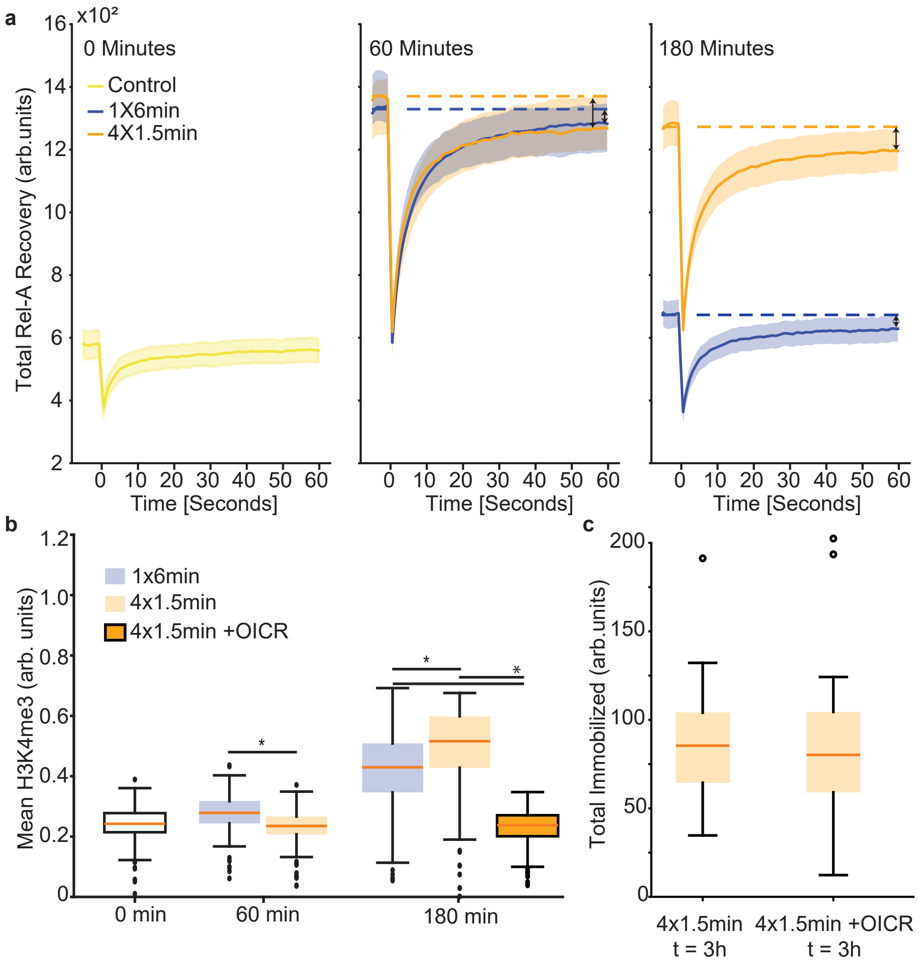

a
x10²
0 Minutes
60 Minutes
180 Minutes
Control
1X6min
4X1.5min
Total Rel-A Recovery (arb.units)
16
14
12
10
8
6
4
2
0 10 20 30 40 50 60
Time [Seconds]
b
Mean H3K4me3 (arb. units)
1x6min
4x1.5min
4x1.5min +OICR
1.2
1.0
0.8
0.6
0.4
0.2
0
0 min
60 min
180 min
c
Total Immobilized (arb.units)
200
150
100
50
0
4x1.5min
t = 3h
4x1.5min +OICR
t = 3h

Supplementary tables

Supplementary Table 1: Summarizes reactions for the D2FC[2] model where "C." and "N." prefixes denote species is in the cytoplasm and nucleus, respectively. The column labeled "name" represents the parameter influencing the reaction rate. The reported values are from the best D2FC[2] model. The rows highlighted in gray represent added model mechanisms that differentiate between D2FC and D2FC[2]. Parameter kv (yellow; value = 3.3) accounts for subcellular compartment volumes between the cytoplasm and nucleus, applied to species traveling between the compartments. DCoop, NPio, NEnh1, NEnh2, and IKKSpots(t) are functions defined in supplementary table 2. Parameter ka (red) was used to optimize the original D2FC model to interface directly with NEMO spot numbers.

Reaction	Name	Value	Rate
$C.IkBa + C.NFkB \rightarrow C.IkBaNFkB$	ka1a	5.854E-01	$ka1a * [C.IkBa] * [C.NFkB]$
$C.IkBaNFkB \rightarrow C.IkBa + C.NFkB$	kd1a	5.000E-02	$kd1a * [C.IkBaNFkB]$
$C.NFkB \rightarrow N.NFkB$	ki1	5.403E-03	$kv * ki1 * [C.NFkB]$
$N.NFkB \rightarrow C.NFkB$	ke1	9.657E-05	$kv * ke1 * [N.NFkB]$
$N.NFkB + N.IkBa \rightarrow N.IkBaNFkB$	f_ka1a	4.25E-02	$f\_ka1a * ka1a * [N.NFkB] * [N.IkBa]$
$N.IkBaNFkB \rightarrow N.NFkB + N.IkBa$	f_kd1a	2.625E-02	$f\_kd1a * kd1a * [N.IkBaNFkB]$
$N.NFkB \rightarrow N.NFkBDNA$	ka1d	7.070E-04	$ka1d * DCoop * NPio * [N.NFkB]$
$N.NFkBDNA \rightarrow N.NFkB$	kd1d	6.684E-05	$kd1d * [N.NFkBDNA]$
$N.IkBa + N.NFkBDNA \rightarrow N.IkBaNFkB$	ka2a	1.320E-02	$ka2a * [N.IkBa] * [N.NFkBDNA]$
$N.IkBa \rightarrow \emptyset$	c4a	5.000E-04	$c4a * [N.IkBa]$
$C.IkBa \rightarrow \emptyset$	c4a	5.000E-04	$c4a * [C.IkBa]$
$N.IkBaNFkB \rightarrow C.IkBaNFkB$	ke2a	8.353E-02	$kv * ke2a * [N.IkBaNFkB]$
$C.IkBa \rightarrow N.IkBa$	ki3a	6.638E-03	$kv * ki3a * [C.IkBa]$
$N.IkBa \rightarrow C.IkBa$	ke3a	2.457E-03	$kv * ke3a * [N.IkBa]$
$C.IkBaNFkB \rightarrow C.NFkB$	c5a	2.200E-05	$c5a * [C.IkBaNFkB]$
$C.IKK \rightarrow C.IKKi$	ki	1.156E-02	$ki * [C.IKK]$
$C.IKKi \rightarrow C.IKKn$	kp	6.464E-05	$kp * [C.IKKi] * A20I$
$C.IKKn \rightarrow C.IKK$	ka	2.409E-06	$\mathrm{TR} * \mathrm{ka} * \mathrm{IKKSpots(t)} * [C.IKKn]$
$\emptyset \rightarrow C.tIkBa$	rs_a	6.852E-08	$rs\_a * (1 + NEnh1)$
$C.tIkBa \rightarrow \emptyset$	c3a	3.000E-04	$c3a * [C.tIkBa]$
$\emptyset \rightarrow C.IkBa$	c2a	5.000E-01	$c2a * C.tIkBa$
$\emptyset \rightarrow C.tCompetitor$	rs_a	6.852E-08	$rs\_a * c1a * NEnh2$
$C.tCompetitor \rightarrow \emptyset$	c6a	4.290E-05	$c6a * [C.tCompetitor]$
$\emptyset \rightarrow C.Competitor$	c2a	5.000E-01	$c2a * [C.tCompetitor]$
$C.Competitor \rightarrow \emptyset$	c4a	5.000E-04	$c4a * [C.Competitor]$
$\emptyset \rightarrow C.tA20$	c1	2.000E-07	$c1 * NEen3$
$C.tA20 \rightarrow \emptyset$	c3	4.000E-04	$c3 * [C.Competitor]$
$\emptyset \rightarrow C.A20$	c2	5.000E-01	$c2 * [C.A20]$
$C.A20 \rightarrow \emptyset$	c4	4.500E-03	$c4 * [C.A20]$
$C.IkBa \rightarrow C.pIkBa$	kc1a	4.998E-01	$kc1a * [C.IKK] * [C.IkBa]$
$C.pIkBa \rightarrow \emptyset$	kt1a	5.796E-03	$kt1a * [C.pIkBa]$
$C.IkBaNFkB \rightarrow pIkBaNFkB$	kc2a	4.071E-01	$kc2a * [C.IKK] * [C.IkBaNFkB]$
$C.pIkBaNFkB \rightarrow C.NFkB$	kt2a	1.274E-04	$kt2a * [C.pIkBaNFkB]$

Supplementary Table 2: Algebraic equations used in the ordinary differential equations as shown in Supplementary Table 1 and used as outputs to the model.

Name	Equation	Notes
$DCoop$	$\frac{[N.NFkB]^{h2}}{(kdNFkB)^{h2}+[N.NFkB]^{h2}}$	Represents the cooperative effects of NFkB binding to DNA.
$NPio$	$1+ps*\frac{[N.NFkBDNA]^{h3}}{(kdP)^{h3}+[N.NFkBDNA]^{h3}}$	Represents pioneering effect of NFKB can have on increasing the availed sites
$A20I$	$\frac{kbA20}{kbA20+C.A20}$	Decrease rate of inactivated IKK converting to neutral IKK.
IKKSpots(t)	$IKK(t)=\sum_{i=1}^{4}a_i e^{\left[-\left(\frac{(x-b_i)}{c_i}\right)^2\right]}$	Parameters a_i, b_i, and c_i are fit to an experimental IKK spot profile. See supplementary information for complete set of parameters.
$NEnh1$	$c1a*\frac{[N.NFkB]^h}{k^h+[N.NFkB]^h}$	Increased rate of IkB transcription due to NFkB retained in nucleus.
$NEnh2$	$c1a*\frac{\left(\frac{[N.NFkB]}{k}\right)^{h+1}}{1+\left(\frac{[N.NFkB]}{k}\right)^{h+1}+\left(\frac{[Competitor]}{k4}\right)^{h+1}}$	Increased rate of Competitor transcription where the competitor itself can reduce its own rate of transcription.
$NEen3$	$c1*\frac{\left(\frac{[N.NFkB]}{k}\right)^{h+1}}{1+\left(\frac{[N.NFkB]}{k}\right)^{h+1}+\left(\frac{[Competitor]}{k2}\right)^{h+1}}$	Increased rate of A20 transcription deepened upon NFkB nuclear localization and the rate can be reduced by competitor formation.
$N.NFkB_{Total}$	$[N.NFkB]+[N.IkBaNFkB]+[N.NFkBDNA]$	Total amount of NFkB in the nucleus including all forms which include free, bound to IkBa and bound to DNA
NFkB (Fold Change)	$\frac{[N.NFkB_{Total}]}{N.NFkB_{Total}(t=0)}$	NFKB fold change that is directly comparable to the single cell data.

Supplementary Table 3: Parameterizations of the top 10 D2FC^2 models found through particle swarm optimization.

	Top 10 D2FC^2 parameterizations									
Name	1	2	3	4	5	6	7	8	9	10
ka1a	5.854E-01	1.018E+00	2.608E-02	9.137E-01	2.505E-01	6.583E-01	8.256E-02	4.916E-01	2.804E+00	1.209E-01
kd1a	5.000E-02	5.000E-02	5.000E-02	5.000E-02	5.000E-02	5.000E-02	5.000E-02	5.000E-02	5.000E-02	5.000E-02
ki1	5.403E-03	2.576E-02	1.068E-02	1.123E-02	5.843E-03	7.873E-03	7.284E-03	1.025E-02	2.519E-02	3.303E-03
ke1	9.657E-05	2.356E-04	2.576E-04	2.524E-05	1.353E-05	1.599E-05	1.788E-05	5.427E-06	2.994E-05	1.522E-05
f_ka1a	4.250E-02	2.148E-02	2.071E-02	3.878E-02	9.525E-02	1.620E-02	1.156E-02	4.217E-01	9.999E-01	1.340E-02
f_kd1a	2.625E-02	1.000E-02	6.686E-01	1.396E-02	9.097E-02	1.793E-02	3.747E-02	6.843E-01	5.618E-02	9.981E-01
ka1d	7.070E-04	1.147E-02	6.930E-05	4.376E-04	1.353E-04	5.532E-03	5.327E-03	1.122E-03	1.013E-03	1.336E-04
ps	7.951E+03	2.368E+03	6.744E+05	3.681E+05	2.878E+04	9.531E+03	1.154E+04	2.670E+05	1.932E+01	2.816E+05
h3	1.000E+00	1.000E+00	1.000E+00	1.000E+00	1.000E+00	1.000E+00	1.000E+00	1.000E+00	1.000E+00	1.000E+00
kdP	3.162E+00	1.123E+00	1.079E+00	1.691E+00	1.599E+00	3.162E+00	2.968E+00	3.160E+00	1.011E+00	2.797E+00
h2	1.500E+00	2.000E+00	1.641E+00	1.955E+00	1.502E+00	1.685E+00	1.998E+00	1.867E+00	1.501E+00	1.500E+00
kdNFKB	1.034E+00	1.308E+00	3.161E+00	3.096E+00	2.304E+00	2.773E+00	2.087E+00	1.434E+00	1.639E+00	2.174E+00
ka2a	1.320E-02	6.274E-02	2.672E-02	2.208E-02	2.197E-02	9.218E+01	2.695E-02	3.337E-01	1.067E-02	1.011E-02
kd1d	6.684E-05	6.919E-05	1.137E-03	6.429E-05	7.110E-05	2.005E-03	8.860E-05	8.467E-04	6.420E-05	6.551E-05
c4a	5.000E-04	5.000E-04	5.000E-04	5.000E-04	5.000E-04	5.000E-04	5.000E-04	5.000E-04	5.000E-04	5.000E-04
ke2a	8.353E-02	4.194E-02	2.341E-02	9.911E-02	1.361E-02	3.751E-03	8.649E-03	1.177E-02	1.549E-02	4.211E-03
ki3a	6.638E-03	1.907E-03	1.839E-03	6.038E-03	2.181E-03	1.318E-03	1.358E-03	5.267E-03	1.750E-03	6.700E-03
ke3a	2.457E-03	1.758E-03	7.948E-04	9.293E-04	1.906E-04	1.948E-04	6.694E-04	3.307E-03	3.572E-05	9.766E-04
c5a	2.200E-05	2.200E-05	2.200E-05	2.200E-05	2.200E-05	2.200E-05	2.200E-05	2.200E-05	2.200E-05	2.200E-05
ka	2.409E-06	2.263E-06	2.595E-05	2.120E-06	9.491E-06	3.078E-06	1.556E-05	2.706E-06	2.238E-06	9.295E-06
ki	1.156E-02	9.935E-03	1.130E-02	1.129E-02	1.138E-02	1.157E-02	1.160E-02	9.715E-03	4.903E-03	1.160E-02
kp	6.464E-05	7.738E-05	2.674E-03	9.060E-05	2.421E-03	1.016E-03	1.370E-03	6.441E-05	1.138E-02	8.211E-03
kbA20	1.800E-03	1.800E-03	1.800E-03	1.800E-03	1.800E-03	1.800E-03	1.800E-03	1.800E-03	1.800E-03	1.800E-03
c1a	1.525E+01	6.487E+00	2.227E+01	8.655E+00	1.245E+01	7.777E+00	2.158E+01	1.415E+01	1.421E+01	2.499E+01
h	2.000E+00	2.000E+00	2.000E+00	2.000E+00	2.000E+00	2.000E+00	2.000E+00	2.000E+00	2.000E+00	2.000E+00
k	6.681E-02	6.515E-02	7.179E-02	6.586E-02	1.263E-01	2.033E-01	9.796E-02	6.501E-02	3.657E-01	6.579E-02
rs_a	6.852E-08	9.820E-08	8.706E-08	6.178E-08	5.182E-08	5.435E-08	5.616E-08	5.001E-08	6.515E-08	7.825E-08
c3a	3.000E-04	3.000E-04	3.000E-04	3.000E-04	3.000E-04	3.000E-04	3.000E-04	3.000E-04	3.000E-04	3.000E-04
c2a	5.000E-01	5.000E-01	5.000E-01	5.000E-01	5.000E-01	5.000E-01	5.000E-01	5.000E-01	5.000E-01	5.000E-01
k4	1.018E-02	1.155E-02	1.479E-01	1.228E-02	2.641E-02	1.842E-01	9.652E-01	8.229E-02	2.209E-01	2.563E-02
c6a	4.290E-05	4.290E-05	4.290E-05	4.290E-05	4.290E-05	4.290E-05	4.290E-05	4.290E-05	4.290E-05	4.290E-05
c1	2.000E-07	2.000E-07	2.000E-07	2.000E-07	2.000E-07	2.000E-07	2.000E-07	2.000E-07	2.000E-07	2.000E-07
k2	9.979E-01	5.847E-01	1.847E-01	5.772E-01	3.202E-02	2.653E-02	1.675E-01	9.754E-01	1.171E-02	1.050E-01
c3	4.000E-04	4.000E-04	4.000E-04	4.000E-04	4.000E-04	4.000E-04	4.000E-04	4.000E-04	4.000E-04	4.000E-04
c2	5.000E-01	5.000E-01	5.000E-01	5.000E-01	5.000E-01	5.000E-01	5.000E-01	5.000E-01	5.000E-01	5.000E-01
c4	4.500E-03	4.500E-03	4.500E-03	4.500E-03	4.500E-03	4.500E-03	4.500E-03	4.500E-03	4.500E-03	4.500E-03
kc1a	4.998E-01	3.603E-01	6.169E-02	4.990E-01	1.010E-01	2.603E-01	1.095E-01	3.514E-01	8.498E-02	2.791E-01
kt1a	5.796E-03	2.681E-05	9.794E-05	1.160E-06	3.056E-03	1.410E-06	2.146E-06	9.194E-04	5.631E-03	6.875E-06
kc2a	4.071E-01	2.305E-01	9.433E-03	2.774E-01	3.883E-02	3.025E-02	1.465E-02	2.703E-01	9.620E-02	1.971E-02
kt2a	1.274E-04	1.760E-04	3.179E-05	1.616E-04	5.877E-05	7.688E-05	2.662E-05	2.133E-04	3.127E-04	3.724E-05

Supplementary Table 4: Prior boundaries of parameters used for particle swarm optimization of the D2FC and D2FC^2 models. D2FC values of N/A represent parameters that were introduced in the D2FC^2 model. High and low parameter bounds are equal for parameter values that were held constant during optimization.

			Particle Swarm Parameter Bounds	
Name	D2FC Value	Units	Low	High
ka1a	0.5	1/(uM*sec)	1.00E-02	1.00E+02
kd1a	0.05	1/sec	0.05	0.05
ki1	0.0026	1/sec	0.00026	0.026
ke1	5.20E-05	1/sec	5.20E-06	5.20E-04
f_ka1a	N/A	dimensionless	0.01	1
f_kd1a	N/A	dimensionless	0.01	1
ka1d	N/A	1/sec	6.42E-05	1.16E-02
ps	N/A	dimensionless	1	1.00E+06
h3	N/A	dimensionless	1	1
kdP	N/A	uM/sec	1	3.1623
h2	N/A	dimensionless	1.5	2
kdNFKB	N/A	uM/sec	1	3.1623
ka2a	N/A	1/(uM *sec)	1.00E-02	1.00E+02
kd1d	N/A	1/sec	6.42E-05	1.16E-02
c4a	0.0005	1/sec	0.0005	0.0005
ke2a	0.01	1/sec	1.00E-03	1.00E-01
ki3a	0.00067	1/sec	6.70E-05	6.70E-03
ke3a	0.000335	1/sec	3.35E-05	3.35E-03
c5a	2.20E-05	1/sec	2.20E-05	2.20E-05
ka	2.00E-06	1/sec	1.00E-07	1.00E-04
ki	0.003	1/sec	6.42E-05	1.16E-02
kp	0.0006	1/sec	6.42E-05	1.16E-02
kbA20	0.0018	uM	0.0018	0.0018
c1a	1.00E+00	dimensionless	1.00E+00	2.50E+01
h	2	dimensionless	2.00E+00	2.00E+00
k	0.065	uM	6.50E-02	3.00E+00
rs_a	3.08E-06	uM/sec	5.00E-08	1.00E-07
c3a	0.0003	1/sec	0.0003	0.0003
c2a	0.5	1/sec	5.00E-01	5.00E-01
k4	0.065	uM	1.00E-02	1
c6a	4.29E-05	1/sec	4.29E-05	4.29E-05
c1	2.00E-07	uM /sec	2.00E-07	2.00E-07
k2	0.065	uM	1.00E-02	1
c3	0.0004	1/sec	0.0004	0.0004
c2	0.5	1/sec	0.5	0.5
c4	0.0045	1/sec	0.0045	0.0045
kc1a	0.074	1/(uM*sec)	0.009	0.5
kt1a	0.1	1/sec	1.00E-06	0.006
kc2a	0.37	1/(uM *sec)	0.009	0.5
kt2a	0.1	1/sec	1.00E-06	0.006

Supplementary Table 5: The reactions, parameter names and rates specific to the IκBβ feedback mechanism added to the D2FC model.

Reaction	Name	Rate
$C.IkBb + C.NFkB \rightarrow C.IkBbNFkB$	ka1b	$ka1b * [C.IkBb] * [C.NFkB]$
$C.IkBbNFkB \rightarrow C.IkBb + C.NFkB$	kd1b	$kd1b * [C.IkBbNFkB]$
$N.NFkB + N.IkBb \rightarrow N.IkBeNFkB$	f_ka1b	$f\_ka1b * ka1b * [N.NFkB] * [N.IkBb]$
$N.IkBbNFkB \rightarrow N.NFkB + N.IkBb$	f_kd1b	$f\_kd1b * kd1b * [N.IkBbNFkB]$
$N.IkBbNFkB \rightarrow C.IkBbNFkB$	ke2b	$kv * ke2b * [N.IkBbNFkB]$
$C.IkBb \rightarrow null$	c4b	$c4b * [C.IkBb]$
$C.IkBb \rightarrow N.IkBb$	ki3b	$kv * ki3b * [C.IkBb]$
$N.IkBb \rightarrow C.IkBb$	ke3b	$kv * ke3b * [N.IkBb]$
$C.IkBbNFkB \rightarrow C.NFkB$	c5b	$c5b * [C.IkBbNFkB]$
$\emptyset \rightarrow C.tIkBb$	rs_b	rs_b
$C.tIkBb \rightarrow \emptyset$	c3b	$c3b * [C.tIkBb]$
$\emptyset \rightarrow C.IkBb$	c2b	$c2b * [C.tIkBb]$
$C.IkBb \rightarrow C.pIkBb$	kc1b	$kc1b * [C.IKK] * [C.IkBb]$
$C.pIkBb \rightarrow \emptyset$	kt1b	$kt1b * [C.pIkBb]$
$C.IkBbNFkB \rightarrow pIkBbNFkB$	kc2b	$kc2b * [C.IKK] * [C.IkBeNFkB]$
$C.pIkBbNFkB \rightarrow C.NFkB$	kt2b	$kt2b * [C.pIkBbNFkB]$

Supplementary Table 6: The reactions, parameters names and rates specific to the IκBε feedback mechanism added to the D2FC model.

Reaction	Name	Rate
$C.IkBe + C.NFkB \rightarrow C.IkBeNFkB$	ka1e	$ka1e * [C.IkBe] * [C.NFkB]$
$C.IkBeNFkB \rightarrow C.IkBe + C.NFkB$	kd1e	$kd1e * [C.IkBeNFkB]$
$N.NFkB + N.IkBe \rightarrow N.IkBeNFkB$	f_ka1e	$f\_ka1e * ka1e * [N.NFkB] * [N.IkBe]$
$N.IkBeNFkB \rightarrow N.NFkB + N.IkBe$	f_kd1e	$f\_kd1e * kd1e * [N.IkBeNFkB]$
$N.IkBeNFkB \rightarrow C.IkBeNFkB$	ke2e	$kv * ke2e * [N.IkBeNFkB]$
$C.IkBe \rightarrow null$	c4e	$c4e * [C.IkBe]$
$C.IkBe \rightarrow N.IkBe$	ki3e	$kv * ki3e * [C.IkBe]$
$N.IkBe \rightarrow C.IkBe$	ke3e	$kv * ke3e * [N.IkBe]$
$C.IkB\epsilon NFkB \rightarrow C.NFkB$	c5a	$c5a * [C.IkB\epsilon NFkB]$
$\emptyset \rightarrow C.tIkBe$	rs_e	$\frac{t^{n_{delay}}}{t^{n_{delay}} + K_{delay}^{n_{delay}}} * rs\_e * (1 + NEnh1)$
$C.tIkBe \rightarrow \emptyset$	c3e	$c3e * [C.tIkBe]$
$\emptyset \rightarrow C.IkBe$	c2e	$c2e * [C.tIkBe]$
$C.IkBe \rightarrow C.pIkBe$	kc1e	$kc1e * [C.IKK] * [C.IkBe]$
$C.pIkBe \rightarrow \emptyset$	kt1e	$kt1e * [C.pIkBe]$
$C.IkBeNFkB \rightarrow pIkBeNFkB$	kc2e	$kc2e * [C.IKK] * [C.IkBeNFkB]$
$C.pIkBeNFkB \rightarrow C.NFkB$	kt2e	$kt2e * [C.pIkBeNFkB]$

Supplementary Table 7: Additional prior boundaries of parameters used for particle swarm optimization of the D2FC + IκBβ model**.** High and low parameter bounds are equal for parameter values that were held constant during optimization.

		Particle Swarm Parameter Bounds	
Name	Units	Low	High
rs_b	micromole/second	5.00E-08	1.00E-07
c3b	1/second	0.0003	0.0003
c2b	1/second	5.00E-01	5.00E-01
c4b	1/second	0.0005	0.0005
ki3b	1/second	3.00E-05	3.00E-03
ke3b	1/second	2.00E-05	2.00E-03
ka1b	1/(micromole*second)	1.00E-02	1.00E+02
kd1b	1/second	0.05	0.05
f_ka1b	dimensionless	0.01	1
f_kd1b	dimensionless	0.01	1
c5b	1/second	2.20E-05	2.20E-05
ke2b	1/second	7.10E-04	7.10E-02
kc1b	1/(micromole*second)	0.009	0.5
kt1b	1/second	1.00E-06	0.006
kc2b	1/(micromole*second)	0.009	0.5
kt2b	1/second	1.00E-06	0.006

Supplementary Table 8: Additional prior boundaries of parameters used for particle swarm optimization of the D2FC + IκBε model**.** High and low parameter bounds are equal for parameter values that were held constant during optimization.

		Particle Swarm Parameter Bounds	
Name	Units	Low	High
rs_e	micromole/second	5.00E-08	1.00E-07
c3e	1/second	0.0003	0.0003
c2e	1/second	5.00E-01	5.00E-01
c4e	1/second	0.0005	0.0005
ki3e	1/second	3.00E-05	3.00E-03
ke3e	1/second	2.00E-05	2.00E-03
ka1e	1/(micromole*second)	1.00E-02	1.00E+02
kd1e	1/second	0.05	0.05
f_ka1e	dimensionless	0.01	1
f_kd1e	dimensionless	0.01	1
c5e	1/second	2.20E-05	2.20E-05
ke2e	1/second	7.10E-04	7.10E-02
kc1e	1/(micromole*second)	0.009	0.5
kt1e	1/second	1.00E-06	0.006
kc2e	1/(micromole*second)	0.009	0.5
kt2e	1/second	1.00E-06	0.006
K_{delay}	seconds	2700	2700
n_{delay}	dimensionless	3	10
