# Supplementary material for: Time-varying stimuli that prolong IKK activation promote nuclear remodeling and mechanistic switching of NF-κB dynamics
Source: Nat Commun. 2025 Aug 8;16:7329. doi: 10.1038/s41467-025-62837-0 (PMC12334592; doi:10.1038/s41467-025-62837-0)
Supplement: Supplementary file 2 — Description of Additional Supplementary Files [file 41467_2025_62837_MOESM2_ESM.pdf]

Description of Additional Supplementary Files:

Supplementary Data 1: STL file for Y-channel microfluidic device. The included file can be 3D printed and used as a mold to produce the PDMS-based microfluidic device used in this study.

Supplementary Data 2: D2FC2 model files. The included model files are used for D2FC2 and related simulations. See also the lab GitHub page for most up to date information (https://github.com/recleelab/D2FCSquared/)

Supplementary Movie 1: Time-lapse images of CRISPR-modified U2OS cells. Fluorescent protein fusions at their endogenous loci are shown EGFP-NEMO (left) and mCherry-RelA (right). 60x images captured the response of two cells exposed to continuous to a saturating 1000 ng/mL of IL-1.

Supplementary Movie 2: Time-lapse images of dual-reporter cells exposed to a single pulse. Dynamics of EGFP-NEMO (left) and mCherry-RelA (right) in response to a single 6- minute pulse of 10 ng/mL IL-1 in the microfluidic device. Cells display typical adaptive behavior within 100 minutes of stimulation.

Supplementary Movie 3: Time-lapse images of dual-reporter cells exposed to four 1.5-minute pulses. Dynamics of EGFP-NEMO (left) and mCherry-RelA (right) in the microfluidic device exposed to 4x1.5-minute pulse of 10 ng/mL IL-1 with 5-minute gaps. Top cells displays prolonged EGFP-NEMO puncta and zero-order export kinetics, whereas the lower cell displays less-sustained EGFP-NEMO puncta and adapts within 180 minutes of stimulation.
