# Supplementary material for: Time-varying stimuli that prolong IKK activation promote nuclear remodeling and mechanistic switching of NF-κB dynamics
Source: Nat Commun. 2025 Aug 8;16:7329. doi: 10.1038/s41467-025-62837-0 (PMC12334592; doi:10.1038/s41467-025-62837-0)
Supplement: Supplementary file 8 — Reporting Summary [file 41467_2025_62837_MOESM8_ESM.pdf]

nature portfolio

Corresponding author(s): Robin E. C. Lee

Last updated by author(s): Jul 14, 2025

Reporting Summary

Nature Portfolio wishes to improve the reproducibility of the work that we publish. This form provides structure for consistency and transparency in reporting. For further information on Nature Portfolio policies, see our Editorial Policies and the Editorial Policy Checklist.

Statistics

For all statistical analyses, confirm that the following items are present in the figure legend, table legend, main text, or Methods section.

n/a	Confirmed	
☐	☒	The exact sample size (*n*) for each experimental group/condition, given as a discrete number and unit of measurement
☐	☒	A statement on whether measurements were taken from distinct samples or whether the same sample was measured repeatedly
☐	☒	The statistical test(s) used AND whether they are one- or two-sided<br>*Only common tests should be described solely by name; describe more complex techniques in the Methods section.*
☐	☒	A description of all covariates tested
☒	☐	A description of any assumptions or corrections, such as tests of normality and adjustment for multiple comparisons
☐	☒	A full description of the statistical parameters including central tendency (e.g. means) or other basic estimates (e.g. regression coefficient) AND variation (e.g. standard deviation) or associated estimates of uncertainty (e.g. confidence intervals)
☐	☒	For null hypothesis testing, the test statistic (e.g. *F*, *t*, *r*) with confidence intervals, effect sizes, degrees of freedom and *P* value noted<br>*Give P values as exact values whenever suitable.*
☒	☐	For Bayesian analysis, information on the choice of priors and Markov chain Monte Carlo settings
☒	☐	For hierarchical and complex designs, identification of the appropriate level for tests and full reporting of outcomes
☐	☒	Estimates of effect sizes (e.g. Cohen's *d*, Pearson's *r*), indicating how they were calculated

Our web collection on statistics for biologists contains articles on many of the points above.

Software and code

Policy information about availability of computer code

Data collection	Matlab 2023b, SimBiology 6.4.1, and provided matlab code. Fusion 360 v2.0.21487 was used to develop CAD files.
Data analysis	Matlab 2023b and Python 3.8

For manuscripts utilizing custom algorithms or software that are central to the research but not yet described in published literature, software must be made available to editors and reviewers. We strongly encourage code deposition in a community repository (e.g. GitHub). See the Nature Portfolio guidelines for submitting code & software for further information.

Data

Policy information about availability of data

All manuscripts must include a data availability statement. This statement should provide the following information, where applicable:

- Accession codes, unique identifiers, or web links for publicly available datasets
- A description of any restrictions on data availability
- For clinical datasets or third party data, please ensure that the statement adheres to our policy

Raw results including single cell trajectory data are included in the Source Data file, and software for analysis are provided as supplementary materials and available at https://github.com/recleelab/D2FCSquared/ and DOI 10.5281/zenodo.15977569.

Data Availability Statement: The single cell time courses and data used for plots generated in this study are provided in the Source Data and Supplementary Data

files.

Research involving human participants, their data, or biological material

Policy information about studies with human participants or human data. See also policy information about sex, gender (identity/presentation), and sexual orientation and race, ethnicity and racism.

Reporting on sex and gender	*Use the terms sex (biological attribute) and gender (shaped by social and cultural circumstances) carefully in order to avoid confusing both terms. Indicate if findings apply to only one sex or gender; describe whether sex and gender were considered in study design; whether sex and/or gender was determined based on self-reporting or assigned and methods used. Provide in the source data disaggregated sex and gender data, where this information has been collected, and if consent has been obtained for sharing of individual-level data; provide overall numbers in this Reporting Summary. Please state if this information has not been collected. Report sex- and gender-based analyses where performed, justify reasons for lack of sex- and gender-based analysis.*
Reporting on race, ethnicity, or other socially relevant groupings	*Please specify the socially constructed or socially relevant categorization variable(s) used in your manuscript and explain why they were used. Please note that such variables should not be used as proxies for other socially constructed/relevant variables (for example, race or ethnicity should not be used as a proxy for socioeconomic status). Provide clear definitions of the relevant terms used, how they were provided (by the participants/respondents, the researchers, or third parties), and the method(s) used to classify people into the different categories (e.g. self-report, census or administrative data, social media data, etc.) Please provide details about how you controlled for confounding variables in your analyses.*
Population characteristics	*Describe the covariate-relevant population characteristics of the human research participants (e.g. age, genotypic information, past and current diagnosis and treatment categories). If you filled out the behavioural & social sciences study design questions and have nothing to add here, write "See above."*
Recruitment	*Describe how participants were recruited. Outline any potential self-selection bias or other biases that may be present and how these are likely to impact results.*
Ethics oversight	*Identify the organization(s) that approved the study protocol.*

Note that full information on the approval of the study protocol must also be provided in the manuscript.

Field-specific reporting

Please select the one below that is the best fit for your research. If you are not sure, read the appropriate sections before making your selection.

☒ Life sciences ☐ Behavioural & social sciences ☐ Ecological, evolutionary & environmental sciences

For a reference copy of the document with all sections, see nature.com/documents/nr-reporting-summary-flat.pdf

Life sciences study design

All studies must disclose on these points even when the disclosure is negative.

Sample size	Single particle tracking experiments for IKK puncta are throughput limited because each movie can only image 2-3 cells. Imaging experiments typically tracked 20 single cells per condition, and exact n-values are reported in figure legends.. Given the input-output nature of our experiments, variability between single cells is a feature that helps establish single-cell input-output trends more broadly.
Data exclusions	For IKK imaging experiments it is essential that the entire cell is visible throughout the movie. Cells that died or partially left the imaging field of view for more than several frames were excluded from analysis. Since the U2OS cells are relatively immobile, there are only a handful of exclusions. Experiments that did not follow the anticipated pattern of Alexa Fluor 647–conjugated BSA in the microfluidic device were rare and were not analysed further as this indicated a failure in either the microfluidic device, or the control system.
Replication	Reproducibility between conditions was evaluated by comparing trends across an array of pulse numbers and gap time durations to establish a consistent trend. The core trend in Figure 2 is a representative of 2 biological replicates. Reproducibility of dynamic cell culture conditions was established by monitoring fluorescence of Alexa Fluor 647–conjugated BSA in the cytokine reservoir.
Randomization	Imaging regions were sampled randomly in the experimental region of the microfluidic device near the inlet channels. Alexa Fluor 647–conjugated BSA was used to confirm that cells were correctly within the experimental region of the device, and to confirm correct operation of the cell culture system.
Blinding	Blinding was not necessary because most of the data analysis relies on automated experimental control systems and semi-automated data extraction

Reporting for specific materials, systems and methods

We require information from authors about some types of materials, experimental systems and methods used in many studies. Here, indicate whether each material, system or method listed is relevant to your study. If you are not sure if a list item applies to your research, read the appropriate section before selecting a response.

Materials & experimental systems

n/a	Involved in the study	
☐	☒	Antibodies
☐	☒	Eukaryotic cell lines
☒	☐	Palaeontology and archaeology
☒	☐	Animals and other organisms
☒	☐	Clinical data
☒	☐	Dual use research of concern
☒	☐	Plants

Methods

n/a	Involved in the study	
☒	☐	ChIP-seq
☒	☐	Flow cytometry
☒	☐	MRI-based neuroimaging

Antibodies

Antibodies used

anti-H3K4me3 antibody (1:100 dilution; Thermo Fisher Scientific, 711958; Lot: 2977681)

Validation

The antibody is verified by Thermo Advanced Verification. Advanced Verification is additional testing that verifies that an antibody will bind to the correct target.

The H3k4me3 antibody was verified by 1) Cell treatment showing reduction via western blot in HeLa cells exposed to Doxorubicin; and 2) detection of enrichment of the targeted histone modification using SNAP-ChIP Spike-in, a proprietary technology developed by EpiCypher, used by Thermo.

Eukaryotic cell lines

Policy information about cell lines and Sex and Gender in Research

Cell line source(s)

U2OS cell lines were purchased from ATCC and CRISPR modified (PMIDs: 30808860 and 34301608). KYM-1 mVen-RelA cells were developed in PMID: 28004761.

Authentication

Research projects have typically focused on this cell line and it is regularly monitored for morphological consistency and expression of the fluorescent reporters. The cell line has not been otherwise authenticated since it was developed.

Mycoplasma contamination

Cell cultures are routinely tested and found negative for mycoplasma contamination on a bi-weekly to monthly basis.

Commonly misidentified lines
(See ICLAC register)

N/A. The cell lines used in this paper do not appear in the list.

Plants

Seed stocks

N/A

Novel plant genotypes

N/A

Authentication

N/A
